# Compensating the Degradation of Near-Infrared Absorption of Black Silicon Caused by Thermal Annealing

**DOI:** 10.1186/s11671-016-1281-4

**Published:** 2016-02-01

**Authors:** Yanchao Wang, Jinsong Gao, Haigui Yang, Xiaoyi Wang, Zhenfeng Shen

**Affiliations:** University of the Chinese Academy of Sciences, Beijing, 100039 China; Key Laboratory of Optical System Advanced Manufacturing Technology, Changchun Institute of Optics, Fine Mechanics and Physics, Chinese Academy of Sciences, Changchun, 130033 China

**Keywords:** Infrared absorption, Thermal annealing, Microstructured silicon, Ag nanoparticles

## Abstract

We propose the use of thin Ag film deposition to remedy the degradation of near-infrared (NIR) absorption of black Si caused by high-temperature thermal annealing. A large amount of random and irregular Ag nanoparticles are formed on the microstructural surface of black Si after Ag film deposition, which compensates the degradation of NIR absorption of black Si caused by thermal annealing. The formation of Ag nanoparticles and their contributions to NIR absorption of black Si are discussed in detail.

## Background

It is well known that Si is the most economical, technologically sophisticated material with the highest crystal quality among all semiconductors. However, due to its wide bandgap of 1.12 eV, Si has less absorption in the near-infrared (NIR) region with the wavelength longer than 1100 nm, which limits its optoelectronic applications within the range of visible to NIR (<1100 nm). Therefore, enhancing the NIR absorption with a longer wavelength has become a topic of great interest. This will extend the photoresponse of Si-based optoelectronic devices into the NIR region (>1100 nm) [1–4]. Recently, a type of Si named “black Si” fabricated by femtosecond (fs)-laser irradiation method has been reported [5, 6]. Black Si exhibits a very high absorption in not only the visible but also the NIR region extending to 2.5 μm because of the special surface microstructure and chalcogen hyperdoping induced by fs-laser processing.

The photodiode on black Si fabricated by fs-laser processing has been demonstrated [7, 8]. It can be even sensitive to the infrared wavelengths up to 1600 nm. Although black Si extends the photoelectric response range to a longer wavelength compared with Si, its photoelectric response intensity in the NIR region is still weak. One possible reason is that its NIR absorption is seriously degraded via thermal annealing process during device fabrication [8]. It was reported that the NIR absorptance from 1200 to 2500 nm decreases to less than 50 % from 90 % after thermal annealing at 775 K due to the diffusion of sulfur ion hyperdoped by fs laser [5]. However, thermal annealing processing associated with ohmic contact formation, defect passivation, or ion activation is a necessary step for photodiode fabrication. Therefore, remedying the degradation of NIR absorption caused by thermal annealing is required in order to improve the NIR photoelectric response of black Si. Newman et al. have attempted to solve the degradation of NIR absorption by annealing at a higher temperature [9]. They showed that the amount of NIR absorption depended on the rate of postannealing cooling, suggesting a kinetically limited deactivation process. In their report, NIR absorption can be reactivated by annealing at a higher temperature between 1350 and 1550 K followed by fast cooling (>50 K/s) because hyperdoped-related defects responsible for NIR absorption are in equilibrium at a higher temperature in black Si.

In this study, we propose the use of thin Ag film deposition to remedy the degradation of NIR absorption of black Si caused by thermal annealing. We characterize the morphology of Ag nanoparticles formed on the microstructure surface after Ag deposition and analyzed its influence on the NIR absorption of black Si by theoretical simulation.

## Methods

Black Si was fabricated on a 450-μm-thick single-side polished boron-doped Si (100) wafer with a resistivity of 10 Ω cm. A 1-kHz, 100-fs, and 800-nm Ti:sapphire laser was used for the laser processing. To fabricate a microstructural surface, cleaned Si wafer was placed on a translation stage in a vacuum chamber filled with high pure SF_6_ gas at a pressure of 5 × 10^4^ Pa. The wafer was irradiated with a snakelike scanning in SF_6_ ambient by the fs laser with a fluence of approximately 0.75 J/cm^2^, leading to the formation of 2 × 2 cm^2^ black Si with a microstructural surface. Then, black Si was thermally treated at 825 K in a furnace with N_2_ ambient for 10 min. Thin Ag films were deposited on the microstructural surface by thermal evaporation at a rate of 0.5 Å/s. The surface morphology was characterized by a scanning electron microscope (SEM). The integrated reflectance (*R*) and transmittance (*T*) spectra between 500 and 2500 nm were measured in a spectrometer (PerkinElmer Lambda-1050) equipped with a 160-mm integrating sphere. The integrated absorptance (*A*) spectra were extracted through *A* = 1 − *R* − *T*.

## Results and Discussion

**Fig. 1:**
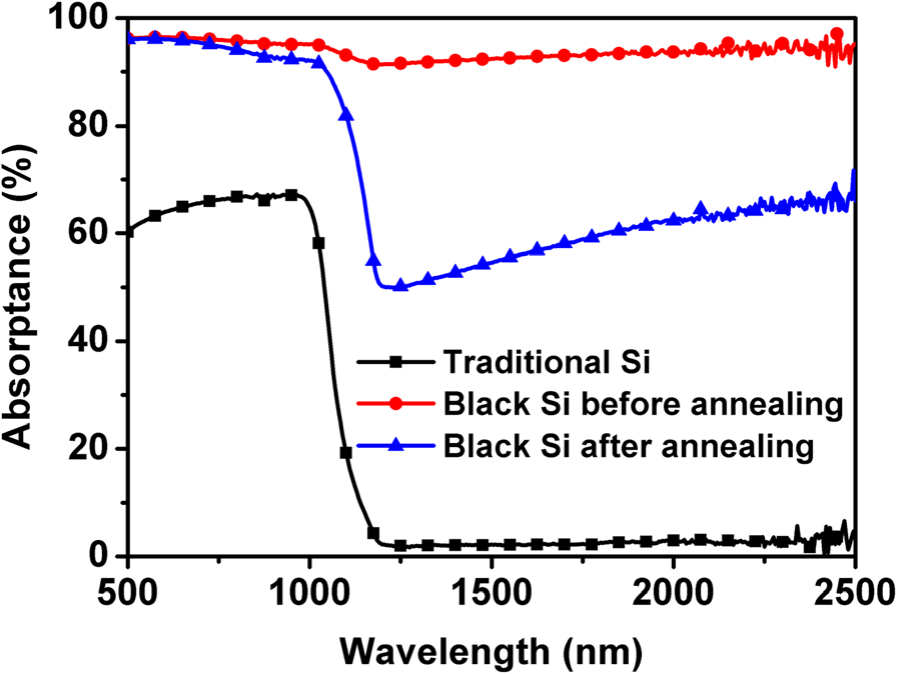
Absorptance spectra of black Si before and after thermal annealing at 825 K, where traditional Si is also given as a comparison

Figure 1 shows the absorptance spectra of black Si before and after thermal annealing at 825 K, where traditional Si is also provided as a comparison. Obviously, traditional Si has less absorption in the NIR region from 1100 to 2500 nm due to its wide bandgap of 1.12 eV. By contrast, black Si exhibits a significant broadband absorption enhancement for the wavelength longer than 1100 nm before annealing, and its average absorptance is higher than 90 %. However, the average absorptance of black Si drastically decreases to approximately 60 % after annealing. Compared with traditional Si, the significant absorption enhancement in the NIR region mainly originates from two factors. One is that fs-laser irradiation induces the formation of an array of spikelike microstructures on black Si surface, which will be presented in the following process. The surface microstructures can trap the light by multiple reflections on the textured surface, which results in an absorption enhancement. The other factor is the S ion hyperdoped by fs-laser irradiation in SF_6_ ambient [5, 6]. The hyperdoping introduces some intermediate bands near the conductance band into the Si bandgap [10], which contributes to below-bandgap absorption and consequently enhances the absorption of black Si in the NIR region. After thermal annealing, NIR absorptance exhibits a drastic decrease. It is suggested that the diffusion of hyperdoping S to the grain boundaries caused by thermal annealing is the reason of NIR absorption degradation [5].

**Fig. 2:**
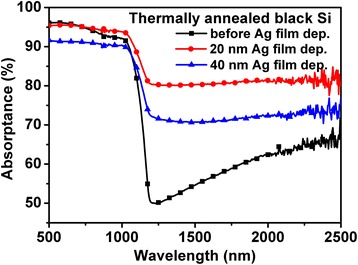
Absorptance spectra of thermally annealed black Si with and without Ag deposition

In order to remedy the absorption degradation, we deposited thin Ag films on the microstructural surface of black Si after thermal annealing. Figure 2 shows the influence of Ag films on the NIR absorption. The sample coated with 20-nm-thick Ag films exhibits the largest broadband absorption enhancement in the whole NIR region from 1100 to 2500 nm, followed by that coated with 40-nm-thick Ag films. The average absorptance increases from 60 to 80 %. Although it does not reach the value of 90 % before annealing shown in Fig. 1, thin Ag films still have a great effect on the absorption recovery.

**Fig. 3:**
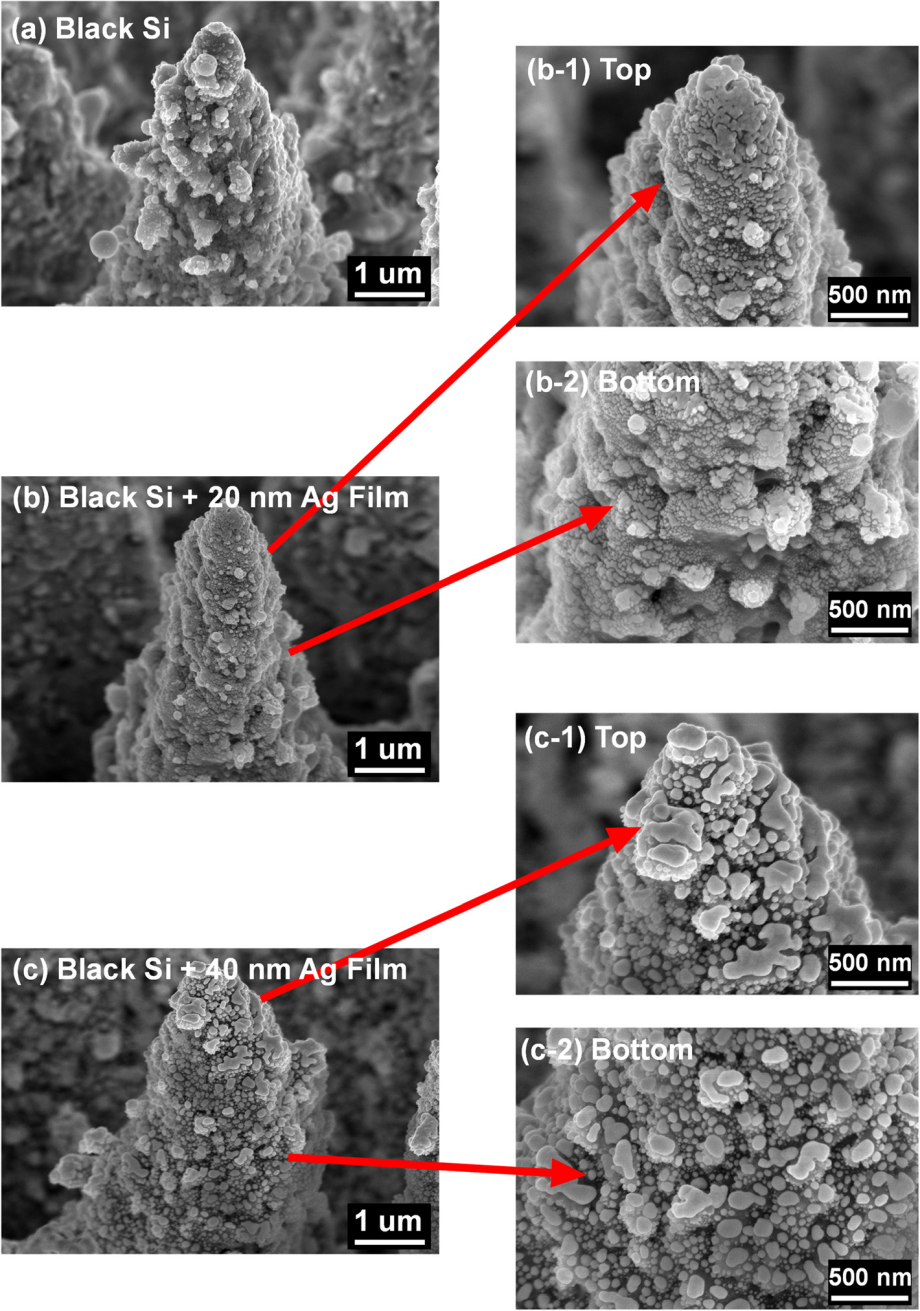
Top-view SEM images at a 45° angle of black Si with and without Ag films(**a**)Black Si (**b**)Black Si with 20nm Ag film dep. (**b-1**)Top position of black Si with 20nm Ag film dep. (**b-2**) Bottom position of black Si with 20nm Ag film dep. (**c**)Black Si with 40nm Ag film dep. (**c-1**)Top position of black Si with 40nm Ag film dep. (**c-2**)Bottom position of black Si with 40nm Ag film dep

To determine why Ag film deposition can remedy the degradation of NIR absorption, we first take the top-view SEM images at a 45° angle of black Si with and without Ag films, as shown in Fig. 3. We can see from Fig. 3a that spikelike microstructures are formed on black Si surface after fs-laser irradiation and furthermore, many nanostructures are also formed on the spikelike microstructural surface. After 20-nm-thick Ag film deposition, from the SEM image in Fig. 3b, especially that with a higher resolution in Fig. 3b-1, b-2, we find that an amount of Ag nanoparticles with irregular shape and different sizes around a few ten nanometers distribute randomly on the microstructural surface. In addition, on the tip positions of micro-/nanostructures of black Si surface, Ag nanoparticles tend to gather together, while they exhibit a scattered and random distribution on the other positions. When it is coated with 40-nm-thick Ag films, Ag particles shown in Fig. 3c grow larger but have a lower density, which might be the origin of higher surface reflection (not shown), resulting in a lower absorption enhancement in Fig. 2 compared with 20-nm-thick Ag films. Figure 3 confirms that Ag atoms deposited on the microstructural surface of black Si by thermal evaporation do not form continuous thin films but an amount of randomly distributed and irregular nanoparticles.

It is known that thin-film kinetic growth is a random deposition process for evaporated materials. With film thickness increasing, continuous films will be gradually developed from discontinuous and island ultrathin films formed at the initial stage of deposition. Generally for Ag film deposition on a Si substrate, we have confirmed that continuous Ag films were formed when their thickness increases to 20 nm. However, in this study, random and irregular nanoparticles instead of continuous films are formed on the microstructure surface of black Si even though the thickness reaches 40 nm, which is shown in Fig. 3. This is mainly caused by the spikelike microstructure surface, resulting in an oblique deposition with a very large deposition angle. In this case, shadowing effect originates from oblique incident atoms being preferentially deposited on the hills of the surface [11–14], leading to the formation of random and irregular particles in Fig. 3b, c. That is also why Ag nanoparticles tend to gather on the tip positions of black Si surface in Fig. 3b, c. In addition, the nanostructures distributed on the spikelike microstructure surface in Fig. 3a further promote the shadowing effect. As a result, an amount of random and irregular Ag nanoparticles are formed even when the deposition thickness reaches 40 nm.

**Fig. 4:**
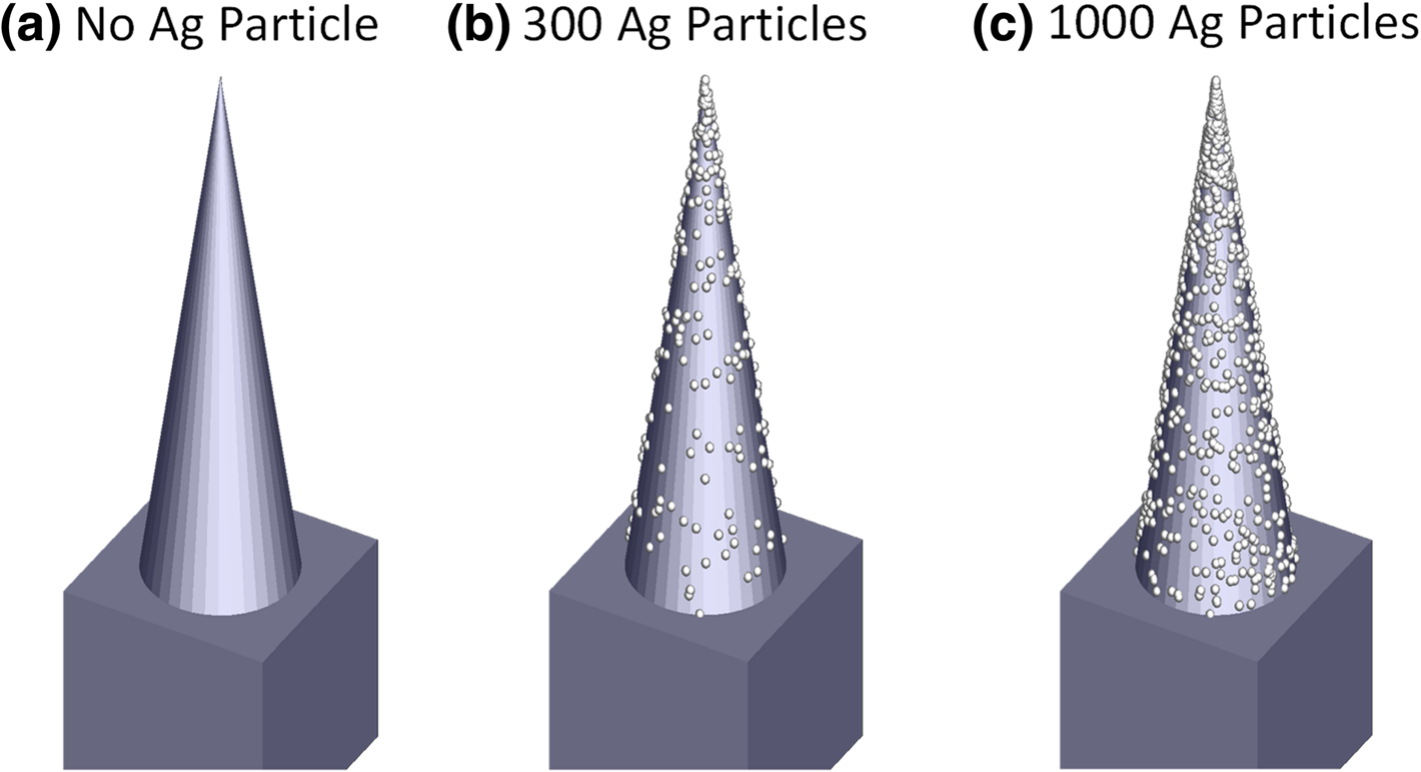
Theoretical models of Si microstructure, **a** without Ag particles and with randomly distributed Ag nanoparticles with the total number of **b** 300 and **c** 1000

**Fig. 5:**
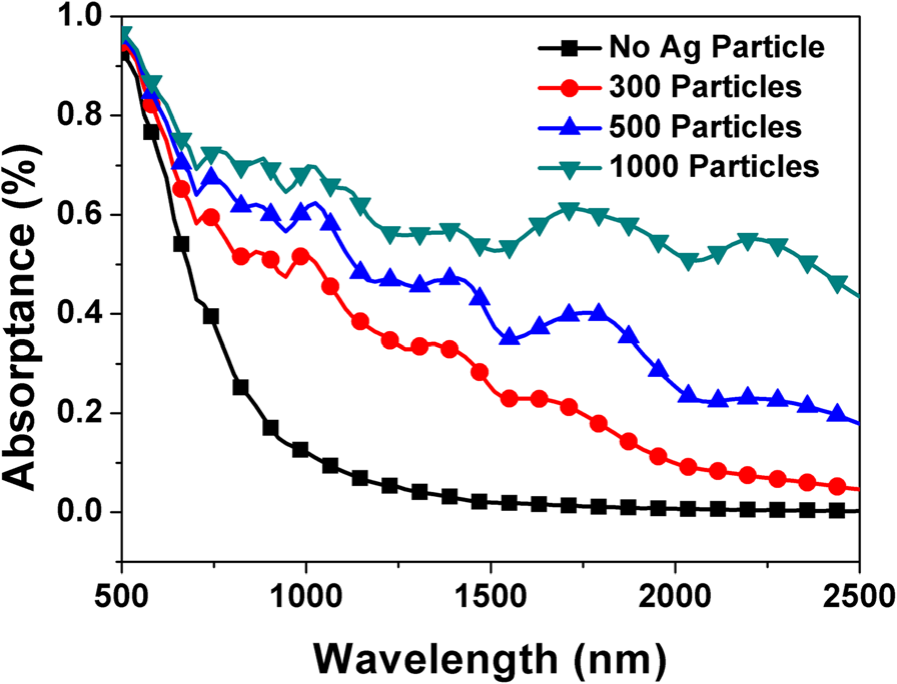
The calculated absorptance without and with Ag nanoparticles

From Fig. 3, it is considered that the formation of randomly distributed Ag nanoparticles remedies the degradation of NIR absorption of black silicon. To determine their physics mechanism, we simulated the effect of Ag nanoparticles on infrared absorption by using a three-dimensional finite-difference time-domain (FDTD) method. The theoretical models are shown in Fig. 4, in which the height and top angle of conical spike are set as 2 μm and 20°, respectively. Ag nanoparticles with the size of 20 nm are randomly distributed onto the microstructure surface. Ag particle number ranges from 300 to 1000. It is noted that we used pure Si material as a substitution body for S-hyperdoped black Si material because it is difficult to extract the optical constants of black Si. When the model with the same shape as the real microstructure (see Fig. 3) but smaller size was determined, we mainly considered two factors. One is that this model can qualitatively reflect the influence of Ag particles on absorption. Another is that the simulation time can be greatly shortened. The size of 20 nm of Ag particle sizes used in the model was determined by a careful observation of the enlarged SEM image of 20-nm-thick Ag film deposition in Fig. 3b-2, from which it was found that Ag particles of several ten nanometers were dominantly distributed on the microstructure surface. Figure 5 shows a comparison of simulated absorptance without and with Ag particles. Obviously, infrared absorption is significantly enhanced in a wide region by randomly distributed Ag nanoparticles. In particular, with the increase of Ag particle number, the absorption enhancement for the longer wavelength region becomes more significant.

**Fig. 6:**
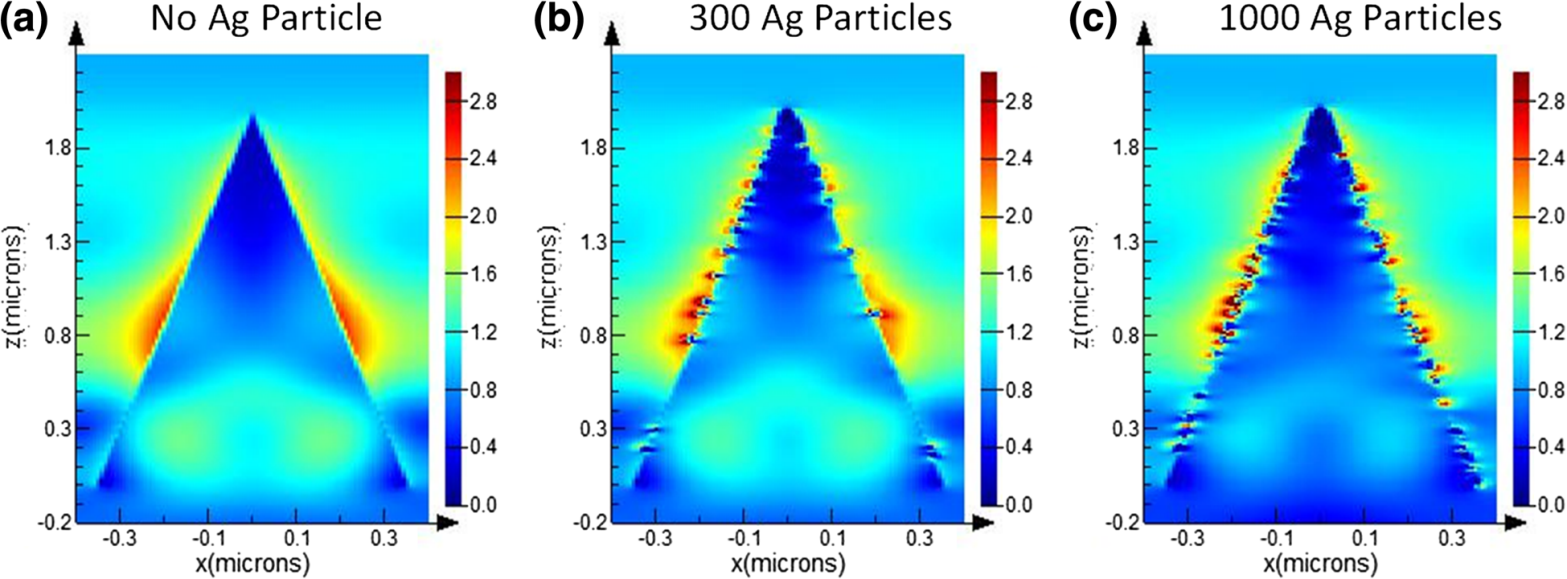
Simulated electric field distribution for the wavelength of 2000 nm of Si microstructure, **a** without Ag particles and with random Ag nanoparticles with the total number of **b** 300 and **c** 1000

The absorption enhancement induced by metal nanoparticles can be attributed to the well-known localized surface plasmon resonance (LSPR). Figure 6 shows the electric field distribution at a cross-sectional direction for the wavelength of 2000 nm. By comparing the electric field without and with Ag particles, we find many hot spots of electric field distributing around Ag nanoparticles. This phenomenon is also observed for other wavelengths from 1200 to 2500 nm (not shown). The strong enhancement of local electrical field intensity is caused by the LSPR induced by the incident light at the interface of Ag particle/Si. Generally, LSPR of sing-type metal nanoparticles leads to a narrowband absorption. However, in this study, broadband absorption enhancement from 1200 to 2500 nm was observed. This should be strongly related to the distribution, the shape, and the size of Ag nanoparticles [15–19]. When the number is set as 300, Ag nanoparticles distributed randomly on the conical spike surface are scattered, which is shown in Fig. 4b. In this case, the probability of Ag particle gathering is relatively low, and therefore, their shape and size have less change. Consequently, the simulated absorption enhancement in Fig. 5 mainly focuses on the shorter wavelength region when Ag particle number is 300. Contrarily, when Ag particle number increases to 1000, as shown in Fig. 4c, the distance between particles shortens, and more and more Ag particles gather together. It leads to the coverage of Ag particles with various shapes and sizes on the conical spike surface, similar to the observed results of SEM images in Fig. 3. As a result, the simulated absorption in Fig. 5 exhibits a broadband enhancement in the whole NIR region from 1100 to 2500 nm, which is consistent quantitively with that in the experimental results. From this viewpoint, we can explain why the absorption by 40-nm-thick Ag film deposition shown in Fig. 2 is lower than that by 20-nm-thick Ag film deposition. By a careful comparison of the enlarged SEM images of 20-nm-thick and 40-nm-thick Ag film deposition in Fig. 3, it is found that the effective particles with a small size around several ten nanometers by 20-nm-thick Ag film deposition are much greater than those by 40-nm-thick Ag film deposition. This is because Ag particles with a small size gather together and become large particles with the increase of Ag film thickness. Therefore, we think that the increase in the number of particles (1000) which resembles the 20-nm-thick Ag film provides a higher absorption, while less amount of particles (300) which is expected to appear after 40-nm-thick Ag film deposition shows less enhancement in absorption. Moreover, large-size particles by 40-nm-thick Ag film deposition cause a higher surface reflection (not shown). As a result, 40-nm-thick Ag film deposition shows a lower absorption compared with 20-nm-thick Ag film deposition.

The proposed method in this study has two main advantages. First, thin Ag film deposition becomes an oblique deposition due to the microstructure surface of black Si, which induces that a large amount of Ag nanoparticles can be conveniently formed on black Si. Second, LSPR generated at Ag particles/Si interface is broadened by the random distribution, the irregular shape, and the different sizes of Ag particles, resulting in a broadband absorption enhancement. It is known that LSPR could enhance the photoelectric response by not only trapping photons [20–22] but also generating hot electrons [9]. Thus, this study provides a promising way to boost the photoelectric response of black Si. However, metal nanoparticles may lead to some negative effects such as local heating, unintended light absorption, and electric field accumulation during biasing the device. Therefore, further studies are required for the contribution of Ag particles to the photoelectric response of black Si.

## Conclusions

We have successfully remedied the degradation of NIR absorption of black Si by using thin Ag film deposition. The average absorptance in the whole NIR region from 1100 to 2500 nm was drastically increased from 60 to 80 % after 20-nm-thick Ag film deposition. Because the conical spike microstructure surface induces an oblique Ag deposition with a very large deposition angle, a large amount of random and irregular Ag nanoparticles with different sizes were formed on the microstructure surface due to the shadowing effect. It contributed to a broadband localized surface plasmon resonance, resulting in a broadband absorption enhancement in the whole NIR region from 1100 to 2500 nm.

## References

[1] Mailoa JP, Akey AJ, Simmons CB, Hutchinson D, Mathews J, Sulivan JT (2014). Room-temperature sub-band gap optoelectronic response of hyperdoped silicon. Nat Commun.

[2] Hu S, Han P, Wang S, Mao X, Li X, Gao L (2012). Improved photoresponse characteristics in Se-doped Si photodiodes fabricated using picosecond pulsed laser mixing. Semicond Sci Tech.

[3] Lin K, Chen H, Lai Y, Yu C (2014). Silicon-based broadband antenna for high responsivity and polarization-insensitive photodetection at telecommunication wavelengths. Nat Commun.

[4] Simmons CB, Akey AJ, Mailoa JP, Recht D, Aziz MJ, Buonassisi T (2014). Enhancing the infrared photoresponse of silicon by controlling the Fermi level location within an impurity band. Adv Funct Mater.

[5] Sheehy MA, Tull BR, Friend CM, Mazur E (2007). Chalcogen doping of silicon via intense femtosecond-laser irradiation. Mater Sci Eng B-Adv.

[6] Peng Y, Zhou Y, Chen X, Zhu Y (2015). The fabrication and characteristic investigation of microstructured silicon with different spike heights. Opt Commun.

[7] Myers RA, Richard F, Karger AM, Carey JE, Mazur E (2006). Enhancing near-infrared avalanche photodiode performance by femtosecond laser microstructuring. Appl Optics.

[8] Carey JE, Crouch CH, Shen M, Mazur E (2005). Visible and near-infrared responsivity of femtosecond-laser microstructured silicon photodiodes. Opt Lett.

[9] Newman BK, Sher MJ, Mazur E, Buonassisi T (2011). Reactivation of sub-bandgap absorption in chalcogen-hyperdoped silicon. Appl Phys Lett.

[10] Shao H, Liang C, Zhu Z, Ning B, Dong X, Ning X (2013). Hybrid functional studies on impurity-concentration-controlled band engineering of chalcogen-hyperdoped silicon. Appl Phys Express.

[11] Karabacak T (2011). Thin-film growth dynamics with shadowing and re-emission effects. J Nanophotonics.

[12] Cansizoglu H, Yurukcu M, Cansizoglu MF, Karabacak T (2015). Investigation of physical vapor deposition techniques of conformal shell coating for core/shell structures by Monte Carlo simulations. Thin Solid Film.

[13] Liu YJ, Chu HY, Zhao YP (2010). Silver nanorod array substrates fabricated by oblique angle deposition: morphological, optical, and SERS characterizations. J Phys Chem C.

[14] He L, Li C, Liu X (2013). The optical properties of alumina films prepared by electron beam evaporation at oblique incidence. Mater Lett.

[15] Mutschke H, Andersen AC, Clément D, Henning T, Peiter G (1999). Infrared properties of SiC particles. Astron Astrophys.

[16] Wang Y, Liu S, Wang Y, Feng G, Zhu J, Zhao L (2009). Infrared light absorption of silver film coated on the surface of femtosecond laser microstructured silicon in SF6. Mater Lett.

[17] Zhang P, Li S, Liu C, Wei X, Wu Z, Jiang Y (2014). Near-infrared optical absorption enhanced in black silicon via Ag nanoparticle-induced localized surface plasmon. Nanoscale Res Lett.

[18] Yang J, Luo F, Kao TS, Li X, Ho GW, Hong M (2014). Design and fabrication of broadband ultralow reflectivity black Si surface by lasers by laser micro/nanoprocessing. Light-Sci Appl.

[19] Tan CL, Jang SJ, Song YM, Alameh K, Lee YT (2014). Bimetallic non-alloyed NPs for improving the broadband optical absorption of thin amorphous silicon substrates. Nanoscale Res Lett.

[20] Wellstood FC, Urbina C, Clarke J (1994). Hot-electron effects in metals. Phys Rev B.

[21] Chalabi H, Schoen D, Brongersma ML (2014). Hot-electron photodetection with a plasmonic nanostripe antenna. Nano Lett.

[22] Chalabi H, Brongersma ML (2013). Harvest season for hot electrons. Nat Nano Technol.

